# Novel Alleles of *gon-2*, a *C*. *elegans* Ortholog of Mammalian TRPM6 and TRPM7, Obtained by Genetic Reversion Screens

**DOI:** 10.1371/journal.pone.0143445

**Published:** 2015-11-25

**Authors:** Eric J. Lambie, Robert D. Bruce, Jeffrey Zielich, Sonia N. Yuen

**Affiliations:** 1 Department of Cell and Developmental Biology, Ludwig Maximilian University, Munich, Germany; 2 Dept. of Internal Medicine, Madigan Army Medical Center, Fort Lewis-McChord, Washington, United States of America; 3 Department of Otolaryngology, Boston Children’s Hospital, Boston, Massachusetts, United States of America; University of Hull, UNITED KINGDOM

## Abstract

TRP (Transient Receptor Potential) cation channels of the TRPM subfamily have been found to be critically important for the regulation of Mg^2+^ homeostasis in both protostomes (e.g., the nematode, *C*. *elegans*, and the insect, *D*. *melanogaster*) and deuterostomes (e.g., humans). Although significant progress has been made toward understanding how the activities of these channels are regulated, there are still major gaps in our understanding of the potential regulatory roles of extensive, evolutionarily conserved, regions of these proteins. The *C*. *elegans* genes, *gon-2*, *gtl-1* and *gtl-2*, encode paralogous TRP cation channel proteins that are similar in sequence and function to human TRPM6 and TRPM7. We isolated fourteen revertants of the missense mutant, *gon-2(q338)*, and these mutations affect nine different residues within GON-2. Since eight of the nine affected residues are situated within regions that have high similarity to human TRPM1,3,6 and 7, these mutations identify sections of these channels that are potentially critical for channel regulation. We also isolated a single mutant allele of *gon-2* during a screen for revertants of the Mg^2+^-hypersensitive phenotype of *gtl-2(-)* mutants. This allele of *gon-2* converts a serine to phenylalanine within the highly conserved TRP domain, and is antimorphic against both *gon-2(+)* and *gtl-1(+)*. Interestingly, others have reported that mutation of the corresponding residue in TRPM7 to glutamate results in deregulated channel activity.

## Introduction

The TRP (Transient Receptor Potential) superfamily of eukaryotic cation channels comprises seven subfamilies: TRPA, TRPC, TRPM, TRPML, TRPN, TRPP and TRPV [1–3]. In all cases, channel activity is thought to result from the association of four subunits, each of which has six transmembrane domains (S1–S6), an S5–S6 pore-lining segment, and cytoplasmic N- and C- termini. The cytoplasmic N and C terminal domains that flank S1–S6 tend to be quite different between different TRP protein subfamilies, with the "TRP domain" (~25 aa immediately after S6) being a notable exception. In some of the TRPM subfamily members, the most C-terminal region has acquired an enzymatic domain: TRPM2 has an ADP-ribose hydrolase domain [4], whereas TRPM6 [5, 6] and TRPM7 [7–9] have an alpha-kinase domain. These domains may play regulatory roles, but enzymatic activity per se is not required for channel activity [10–13].

The functions of different regions of TRP channels have been inferred through a variety of methods, including comparison with previously characterized protein motifs e.g., [4, 7, 14], sequencing of disease-causing alleles, e.g., [15–17], site-directed mutagenesis of candidate regulatory residues, e.g., [18–20], and *in vivo* selection for variants with altered activity,.e.g., [21, 22]. Recently, high resolution structural data have been published for TRPVI [23, 24] and TRPA1 [25], confirming the tetrameric structure prediction and highlighting the importance of the TRP domain for channel gating. The structural data also revealed additional sequences in TRPV1 and/or TRPA1 that are important for the regulation of channel activity, such as the "pre-S1 helix" and adjacent linker sequence, discussed further below. In the case of TRPM channels, relatively low resolution structural data are available for TRPM2, and these suggest extensive interactions between the N- and C-terminal cytoplasmic domains, similar to that reported for TRPA1 [26].

The genome of the nematode, *Caenorhabitis elegans*, contains three paralogous genes, *gon-2*, *gtl-1* and *gtl-2*, which encode cation channel proteins (GON-2, GTL-1 and GTL-2) that are similar to human TRPM6 and TRPM7, but lack the kinase domain. Like TRPM6 and TRPM7, the channels formed by GON-2, GTL-1 and GTL-2 are permeable to the divalent cations, Ca^2+^ and Mg^2+^, and play a role in the systemic regulation of Mg^2+^ that is conserved in humans, Drosophila and nematodes [6, 27–31]. GON-2 and GTL-1 are both expressed in the intestine and act semi-redundantly to mediate the uptake of Mg^2+^ from the gut lumen. *gon-2(lf); gtl-1(0)* (i.e., *gon-2* loss-of-function; *gtl-1* null) animals are unable to grow unless the medium is supplemented with high levels of Mg^2+^. GON-2 probably also functions in the somatic gonad precursor cells, since these cells fail to proliferate in *gon-2(lf)* mutants [32]. GTL-2 is expressed in both the hypodermal cells and the excretory cell and is required for clearance of excess Mg^2+^ via the excretory system [31, 33]. *gtl-2(0)* mutants are unable to grow if the medium is supplemented with high levels of Mg^2+^.

We have performed extensive genetic screens for mutations that suppress the gonadogenesis defect of *gon-2(q388ts)* mutants, and these resulted in the identification two new loci, *gem-1* [34] and *gem-4* [35]. During these screens, we also identified multiple intragenic suppressor mutations within *gon-2*, and these are described in this paper. We have also screened for mutations that suppress the Mg^2+^ sensitive phenotype of *gtl-2(0)* mutants, and this resulted in the identification multiple loss-of-function alleles of *gtl-1* [31], plus an unusual antimorphic allele of *gon-2*, which is described here. Together, these mutations in *gon-2* highlight a series of residues that are potentially important for TRPM channel regulation. In some cases, these are in regions of the protein that have been previously implicated in the regulation of TRP channel activity.

## Materials and Methods

### Nematode culture

*C*. *elegans* culture and genetic manipulations were performed essentially as described previously [31, 32, 34].

### Molecular biology

Standard methods were used to PCR amplify and sequence genomic DNA from *C*. *elegans*.

### Amino acid alignments

The software package CLC Main Workbench v. 6.6.5 (www.clcbio.com) was used to perform amino acid alignments for graphical output.

### Strains

The following strains were used: CB4856 Wild type Hawaiian; LX929 vsIs48[Punc-17::gfp] X; EJ556 gon-2(q388dx60) unc-29(e1072) I; EJ557 gon-2(q388dx65) unc-29(e1072) I; EJ652 unc-13(e51) gon-2(dx87) I; EJ720 gon-2(q388dx96) unc-29(e1072) I; EJ922 gon-2(q388dx116) unc-29(e1072) I; EJ959 gon-2(q388dx99) unc-29(e1072) I; EJ1021 gon-2(q388dx148); gem-1(bc364) I; EJ1110 gon-2(q388) I; EJ1173 vsIs48[Punc-17::gfp] N2/CB4856 hybrid X; EJ1190 gon-2(q388); vsIs48[Punc-17::gfp]; EJ1191 gon-2(q388dx146); gem-1(bc364) I; EJ1192 unc-13(e51) gon-2(ok465)/unc-13(e51) lin-11(n566) I; EJ1193 gon-2(q388dx146); gem-1(bc364) I.

### Dosage testing

Strain LX929 *vsIs48[Punc-17*::*gfp]* X, which expresses GFP in all cholinergic neurons, was obtained from Michael Koelle (Yale University). LX929 (N2) males were mated with wild type CB4856 (Hawaiian) males, and *vsIs48* homozyous hermaphrodites were reisolated from the F2 to generate strain EJ1173. Males of this strain retain the vigorous mating ability of CB4856. EJ1173 males were crossed with *gon-2(q388)* hermaphrodites and *gon-2(q388); vsIs48* hermaphrodites were isolated from the F2 to generate strain EJ1190. These males are also vigorous maters and were used for the dosage experiments as follows. L4-stage hermaphrodites of genotype *gon-2(q388sup)* (i.e., intragenic revertant/suppressor alleles of *gon-2(q388)*) were incubated overnight at 23 C, then transferred to a cross plate containing multiple EJ1190 males. Incubation was continued at 23 C, and adults were transferred to a new plate after approximately 8 hrs, then removed 14 hours later. F1 hermaphrodite progeny were scored when they reached adulthood, with cross progeny uniquely identifiable due to the dominant *vsIs48* marker. Self progeny data were collected in parallel using GFP negative animals and/or plates where no cross was performed. All *gon-2(q388sup)* alleles except *dx116* were outcrossed once or more before testing suppression efficiency.

### Divalent cation depleted media (DCDM)

10 g of Agar (Carl Roth, Kobe I) was washed first with 500 ml of 20 mM EDTA, then three times with deionized water to remove most divalent cations. This was then used to prepare solid growth medium of the following final composition: 2% Agar, 4 mg/ml BactoTryptone (Difco), 25 mM HEPES pH7.4, 25 mM NaCl, 5 micrograms/ml cholesterol. 1M MgSO_4_ and 1M CaCl_2_ stock solutions were added in appropriate amounts for Mg^2+^ and Ca^2+^ supplementation experiments.

### Genetic characterization of *gon-2(dx87)* and comparison with *gon-2(ok465)*

Hermaphrodites of genotype *unc-13(e51) gon-2(ok465)/unc-13(e51) lin-11(n566)* were crossed with EJ1173 males and multiple non-Unc L4-stage hermaphrodites were transferred to DCDM plates and incubated at 23 C. *unc-13(e51) gon-2(ok465)* F2 hermaphrodites (distinguishable due to their sterility) were scored as adults. Essentially the same procedure was followed for *unc-13(e51) gon-2(dx87)/+ +* hermaphrodites, but F1s were not singled since all had the same genotype.

## Results and Discussion

### Reversion of *gon-2(q388)*

**Fig 1:**
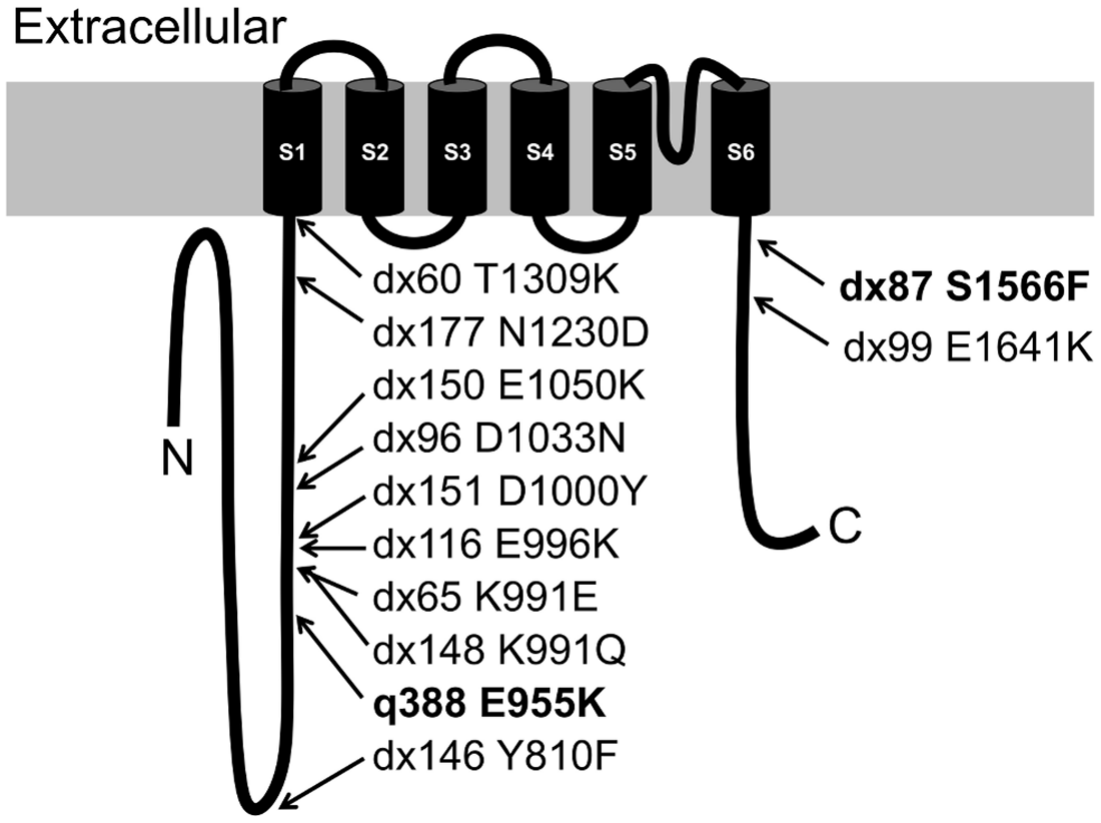
Topology map of GON-2 (2032aa) with locations and aa changes of mutations. The inactivating mutations, *q388* and *dx87*, are shown in bold.

Although we isolated multiple loss-of-function alleles of *gon-2* during our initial characterization of this gene, most of our studies have utilized the *gon-2(q388)* allele [32]. This allele is advantageous, because all animals are fertile when raised at permissive temperature (15°-20°C), but nearly all (>90%) are gonadless (Gon)/sterile when raised at restrictive temperature (> 23°C). In dosage tests, *gon-2(q388)* exhibits very little activity at restrictive temperature. However, *gon-2(q388)* does evidently retain a low level of activity under restrictive conditions, because it can be suppressed by *gem-4(lf)* mutations, unlike the null/deletion allele, *gon-2(ok465)* [35]. *gon-2(q388)* converts a highly-conserved acidic residue (Glu) within the N-terminal cytoplasmic domain of GON-2 to a basic residue (Lys) (Fig 1) [36]. The results of reciprocal temperature shift experiments, suggest that *gon-2(q388)* probably affects a transient, irreversible process that is necessary for GON-2 stability, e.g., protein folding and/or channel assembly [32].

Intragenic revertants of *gon-2(q388ts)* were obtained by selecting for fertile derivatives at restrictive temperature after mutagenizing with EMS or 310 nm UV irradatiation, essentially as described previously [34, 35]. Some screens included the *gem-1(bc364)* mutation, which enhances the penetrance of *gon-2(q388)* [34]. In the case of mutations that were found to be linked to *gon-2* during outcrossing, the *gon-2* coding sequence was PCR amplified and sequenced. The frequency of intragenic revertants was approximately 5 x 10^−4^ per mutagenized genome. We did not find any cases in which the *q388* mutation had directly reverted back to the wild type sequence. Possibly, this is because direct reversion would require a T -> C transition mutation, which is not favored by either EMS or 310 nm UV treatment.

### Genetic characterization of intragenic revertants

The suppression efficiency of representative intragenic revertants was assessed by selfing homozygous *gon-2(q388sup)* hermaphrodites at restrictive temperature and scoring the gonadogenesis phenotype of the progeny. The efficiency of suppression ranges from 85% (*dx99*) to 100% (*dx146*) (Table 1). This group of intragenic revertant mutations was also tested for haplosufficiency by crossing homozygous *gon-2(q388sup)* hermaphrodites with *gon-2(q388)* males at restrictive temperature and scoring the offspring. *gon-2(q388)* has very little activity under these conditions, so this assay provides a good measure of dosage sensitivity of *gon-2(q388sup)* alleles. The strength of suppression among animals heterozygous for the suppressor alleles correlates well with the suppression efficiency measured for the homozygous strains (Table 1). In most cases, suppression efficiency is slightly lower when the intragenic revertant mutation is heterozygous, indicating that these alleles do not fully restore wild type gene function. However, *gon-2(q388dx146)* fully suppresses the gonadogenesis defect of *gon-2(q388)*, even when present in only a single dose.

**Table 1:** Dosage Testing of Intragenic Revertant Mutations.

Allele Configuration	% Vul	% Evul	%WT	n
*gon-2(q388)/gon-2(q388)* ^1^	93	5	2	553
*gon-2(q388dx60)/gon-2(q388dx60)* ^2^	7	4	89	124
*gon-2(q388dx60)/gon-2(q388)*	11	8	81	132
*gon-2(q388dx99)/gon-2(q388dx99)* ^3^	10	5	85	124
*gon-2(q388dx99)/gon-2(q388)*	24	15	51	164
*gon-2(q388dx116)/gon-2(q388dx116)* ^4^	0	0	100	140
*gon-2(q388dx116)/gon-2(q388)*	0	5	95	153
*gon-2(q388dx146)/gon-2(q388dx146)* ^5^	0	0	100	516
*gon-2(q388dx146)/gon-2(q388)*	0	0	100	583
*gon-2(q388dx65)/gon-2(q388dx65)* ^6^	9	1	90	238
*gon-2(q388dx65)/gon-2(q388)*	23	8	69	159
*gon-2(q388dx148)/gon-2(q388dx148)* ^7^	0	0.5	99	223
*gon-2(q388dx148)/gon-2(q388)*	0	1	99	257

Allele configuration is for animals derived from selfing or crosses, as described in Materials and Methods. Full genotypes of parental strains are listed in Materials and Methods. Vulvaless (Vul) animals have a severe gonadogenesis (Gon) phenotype, whereas Everted vulva (Evul) animals have a less severe defect in gonad development. Animals scored as wild type (WT) based on vulva morphology were also usually fertile.

^1^ EJ1190

^2^ EJ556

^3^ EJ959

^4^ EJ922

^5^ EJ1193

^6^ EJ557

^7^ EJ1021

### Molecular characterization of intragenic revertant mutations

The locations of mutant alleles of *gon-2* relative to the predicted protein topology are shown in Fig 1. Most sites were identified by only a single mutation, indicating that the screen is not near saturation. The codon for E996 appears to contain a mutational hotspot, since the identical C -> T mutation was obtained independently as five different alleles, *dx115*, *dx116*, *dx129*, *dx130* and *dx147* (only *dx116* is indicated in Fig 1). Most of the revertant mutations are relatively near *gon-2(q388)* within the N-terminal cytoplasmic domain. Consistent with the idea that this area is preferentially affected, *dx148* (K991Q) and *dx65*(K991E) cause different alterations to a single residue within this region. In each case, it is possible that the intragenic revertant mutation acts by somehow compensating for the effects of the *gon-2(q388)* mutation on protein folding/channel assembly. However, in the descriptions below, we focus on the potential significance of the mutations with regard to effects on channel activity that are independent of the effects *gon-2(q388)*.

#### dx146

**Fig 2:**
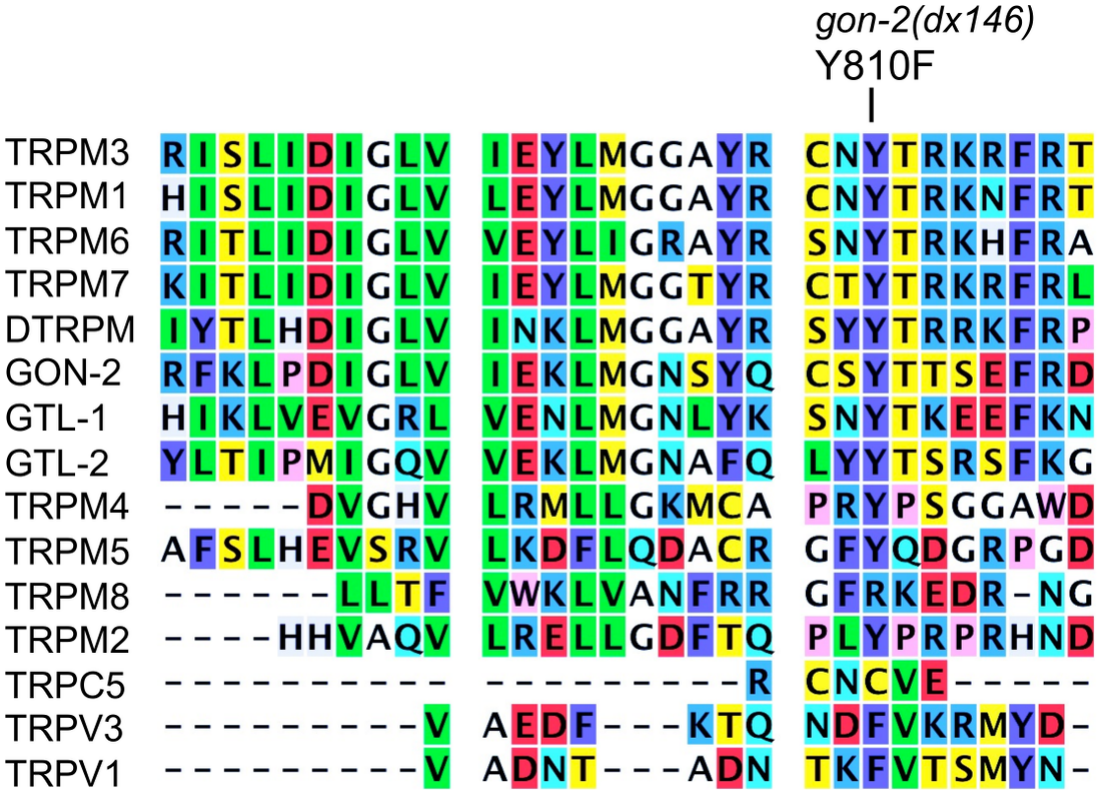
Alignment of representative TRP channel protein sequences near the site of the *gon-2(dx146)* mutation. The following GenBank accession numbers (or UniProt identifiers) were used to retrieve amino acid sequences of proteins shown in alignments. Human proteins: TRPV1 Q8NER1, TRPV3 Q8NET1, TRPC5 Q9UL62, TRPM1 NP_001238949, TRPM2 XP_011528036, TRPM3 NP_060132, TRPM4 NP_060106, TRPM5 NP_055370, TRPM6 NP_060132, TRPM7 NP_060142, TRPM8 NP_076985. *C*. *elegans* proteins: GON-2 CAB02303, GTL-1 CAA92726, GTL-2 CAB00861. *Drosophila melanogaster* protein: DTRPM A8DYE2.

This mutation alters a tyrosine residue that is also found in mammalian TRPM1,3,6,7 and Drosophila TRPM (DTRPM) (Fig 2). This is a candidate Src phosphorylation site YT [37] that could potentially affect channel localization, turnover, or inter-subunit interaction. Notably, the N-terminal cytoplasmic domain of TRPM6 contains multiple ankyrin-like domains that are likely to mediate interaction between channel subunits [38]. For example, the S141L mutation in human TRPM6 affects one of these ankyrin repeats and prevents channel subunit assembly, thus leading to hereditary Mg^2+^ deficiency [39].

#### dx65, dx148

**Fig 3:**
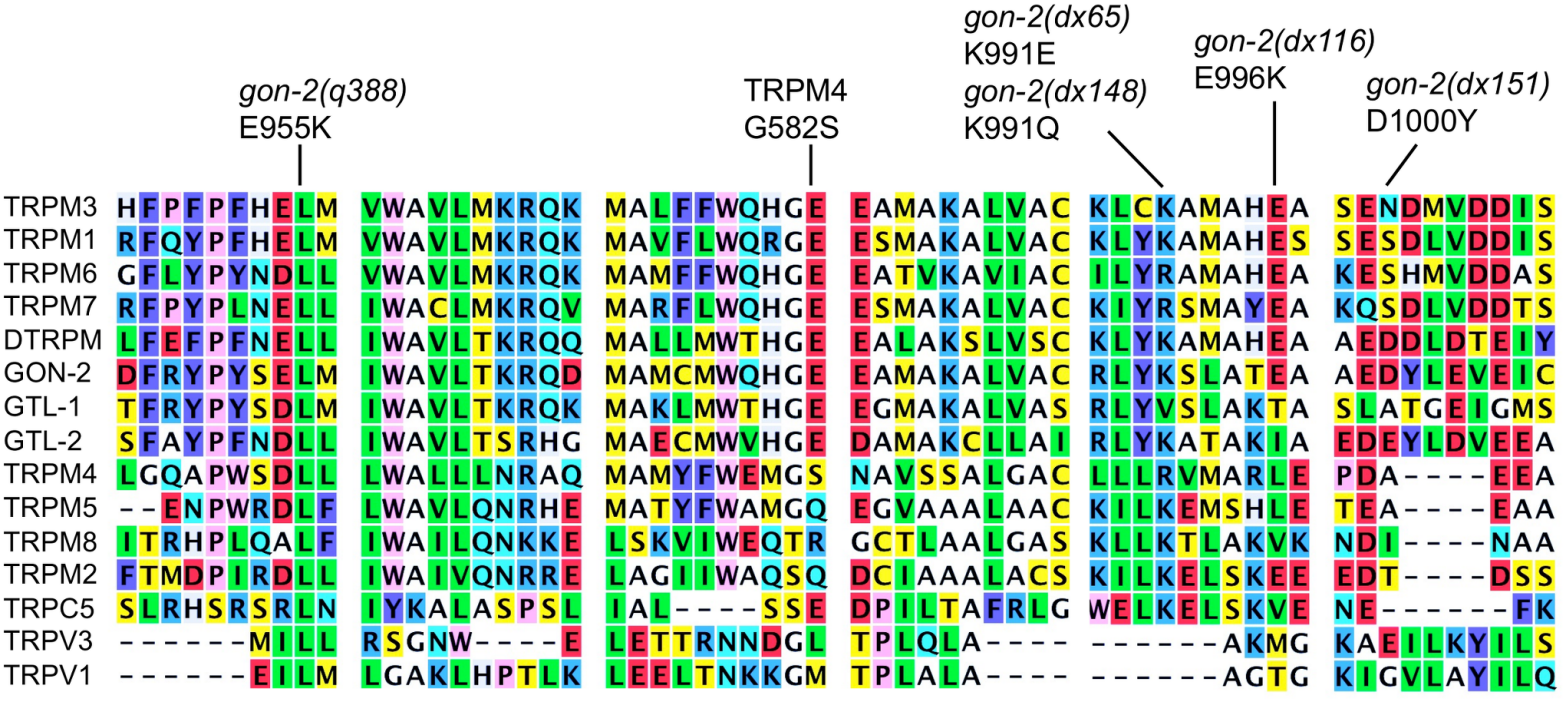
TRP protein alignment from *gon-2(q388)-gon-2(dx151)*.

These mutations both affect the same, highly conserved lysine residue (K991), relatively close to *gon-2(q388)* K955E (Fig 3). Lysines are candidate sumoylation sites, and sumoylation status is known to affect the activity of TRPM proteins [40]. However, this is probably not the basis for the effects of *dx65* and *dx148*, since they produce quantitatively different results (Table 1) even though each eliminates the candidate sumoylation site.

Additionally, of possible significance is the report by Stallmeyer et al. that in certain individuals with a putative gain-of-function mutation in TRPM4, a nearby conserved glycine is converted to serine (G582S; Fig 3) [41]. However, other amino acid alterations within TRPM4 were also present in these individuals, so it is uncertain whether G582S increases, decreases or has no effect on TRPM4 activity.

#### dx116 and dx151

These mutations affect residues immediately downstream of *dx65/dx148* (Fig 3), converting a moderately conserved glutamate to lysine (*dx116*, E996K) and a non-conserved aspartate to tyrosine (D1000Y).

#### dx96 and dx150

**Fig 4:**
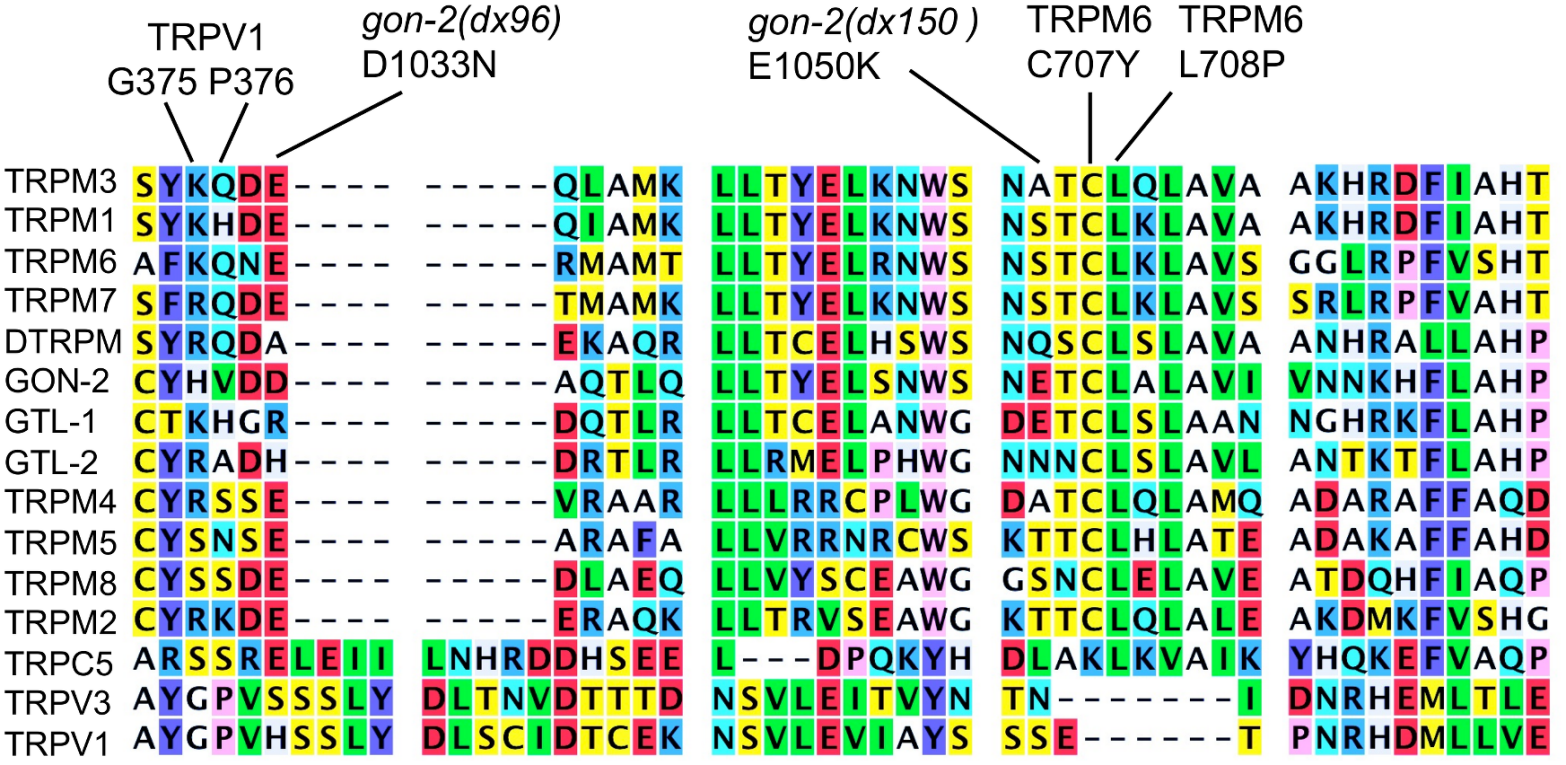
TRP protein alignment from *gon-2(dx96)—gon-2(dx150)*.

These affect a highly conserved acidic residue (*dx96* D1033N) and a non-conserved acidic residue (*dx150* E1050K) within a region that is important for TRPM6 activity (Fig 4). Jalkanen et al. identified the C707Y allele [42] and Lainez et al. identified the L708P allele [43] in patients with hypomagnesemia and secondary hypercalcemia (HSH). Lainez et al. found that the L7808P mutation severely impairs channel activity, but does not affect expression and trafficking, leading them to speculate that this region is required for proper interaction between channel subunits [43]. This region of TRPV1 forms a linker domain that is situated between the N-terminal ankyrin repeats and the pre-S1 helix [24]. Notably, G375 and G376 in TRPV1 mediate direct interaction between the linker domain and the N-terminal ankyrin repeats of ajdacent subunits [24]. The sequence identity between TRPM and TRPV proteins is very low in this region; however, ankyrin-like repeats do exist in the N-terminal cytoplasmic domain of TRPM6 and 7 [38].

#### dx177

This mutation converts an aspargine to aspartate. No alignment is shown because the alteration occurs in a section of GON-2 that has similarity only to GTL-1.

#### dx60

**Fig 5:**
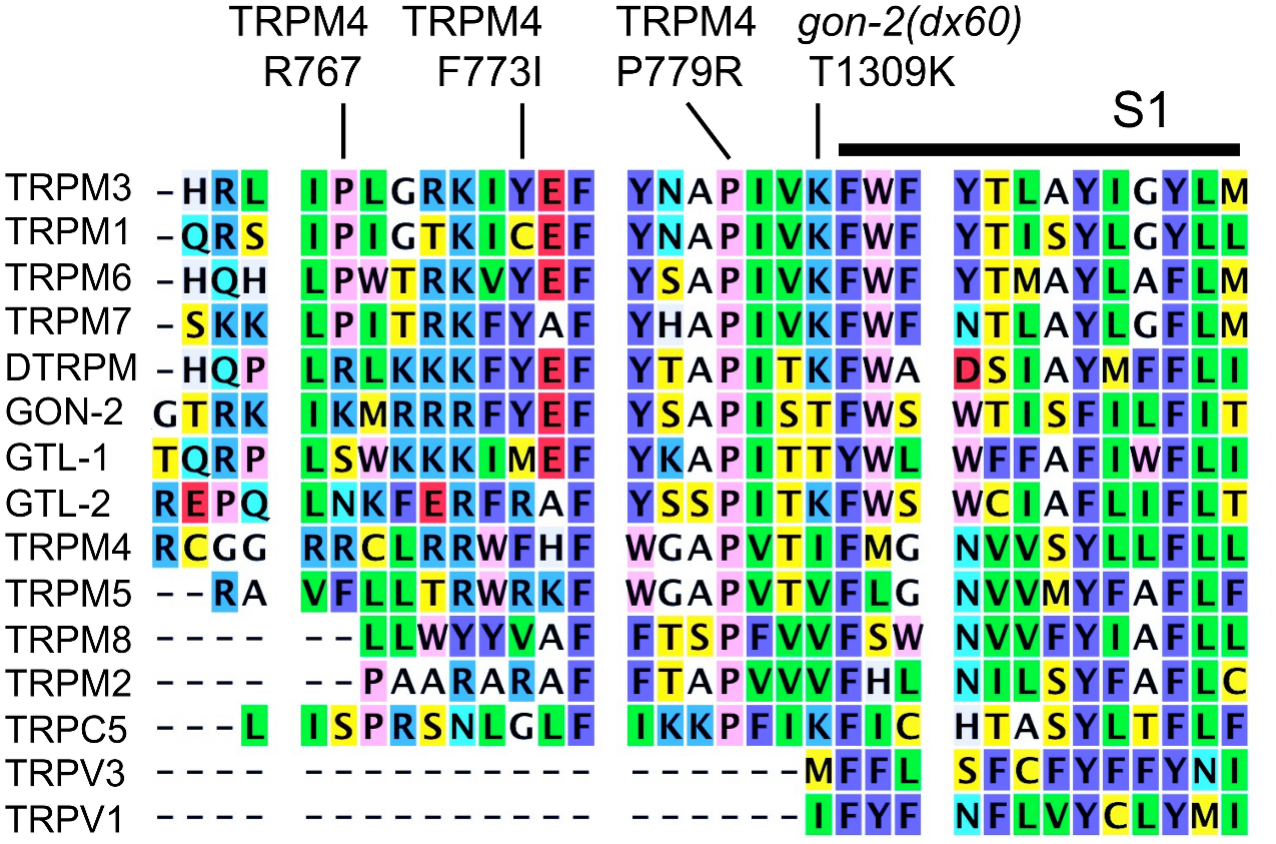
TRP protein alignment near *gon-2(dx60)*.

This mutation converts an atypical threonine to lysine (Fig 5). Interestingly, this appears to represent a return to an ancestral state, since lysine is present at this position in GTL-2, as well as in the most closely related TRPM channels from mammals and Drosophila. *dx60* is situated at the end of the "pre-S1 helix" [24]. In TRPV1, this region interacts directly with with the TRP domain, probably to maintain channel closure. Although this region of TRPM channels is not highly similar to TRPV1, it is predicted to have helical structure [44]. Therefore, *dx60* could potentially render the GON-2 channel more active by increasing its open probability. Of additional possible significance is that fact that pre-S1 helix region of GON-2 contains a sequence (QGTRKKIKMRRRFYEFYSAPI) that is predicted to bind to calmodulin (http://calcium.uhnres.utoronto.ca/ctdb/ctdb/sequence.html). Conceivably, calmodulin could compete with the TRP domain for binding to the pre-S1 helix and this could explain the ability of low levels of cytoplasmic Ca^2+^ to activate GON-2 [45].

In the case of TRPM4, the pre-S1 helix interacts with phosphoinositides that modulate channel gating [46], so this is another protential regulatory mechanism that could be affected by *dx60*.

Also of potential significance, Ubipred[47] predicts that 3 of the 8 high-confidence ubiquitylation sites in GON-2 (K1255, K1269 and K1272) are situated nearby. A decrease in ubiquitlyation efficiency would be expected to result in increased channel expression on the plasma membrane [48–50].

#### dx99

**Fig 6:**
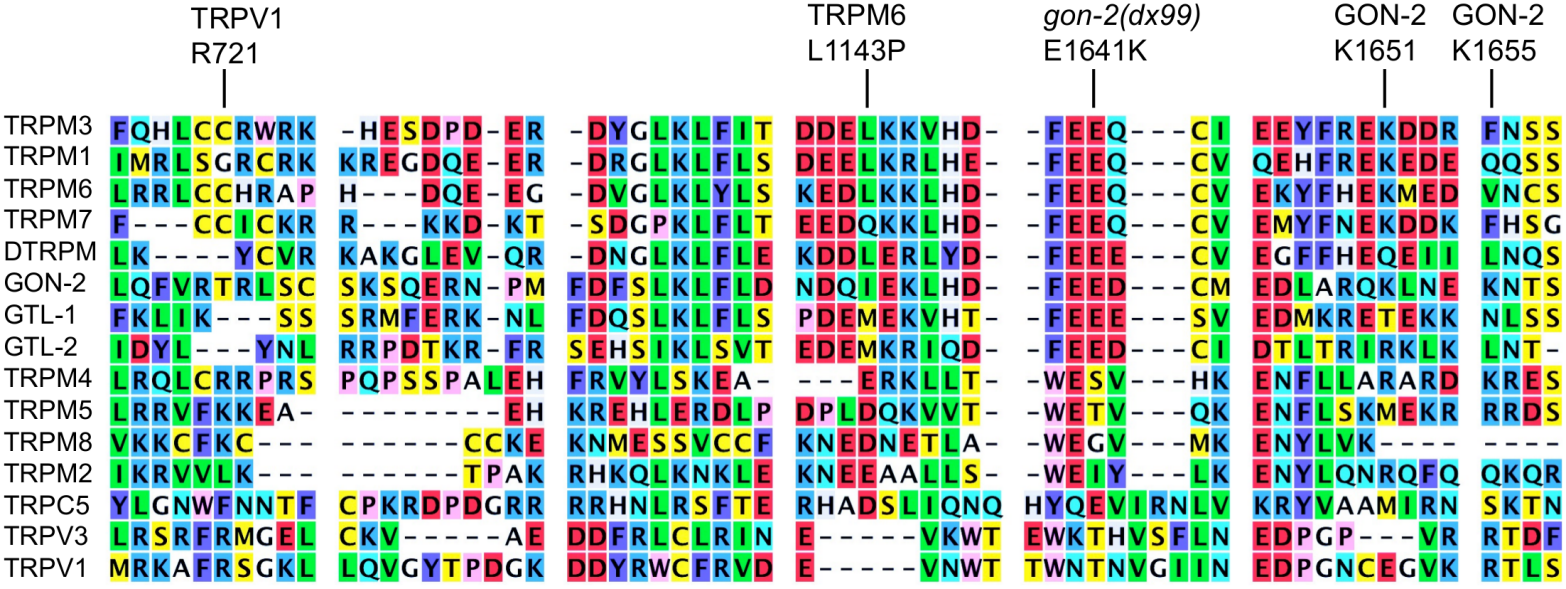
TRP protein alignment near *gon-2(dx99)*.

This mutation converts a moderately conserved glutamate to lysine (Fig 6). This region of the protein is notable for multiple reasons. First, Lainez et al. identified the L1143P mutation of TRPM6 in a patient with HSH [43], and found that while the mutation severely impairs channel activity, it does not affect expression and/or trafficking. Second, two of the eight high-confidence candidate ubiquitylation sites within GON-2 are in this region, K1655 and K1659 [47]. Third, R721 of TRPV1 is required for channel activation by specific phosophoinositides, probably via direct interaction [51]. The corresponding residue within GON-2 is situated within a high-confidence calmodulin binding sequence (LYHGVLILQFVRTRLSCSKS; http://calcium.uhnres.utoronto.ca/ctdb/ctdb/sequence.html).

Several studies suggest that this region of GON-2 is likely to mediate direct interaction between channel subunits via the formation of coiled coils. Mei et al., showed that a predicted coiled-coil forming region of TRPM2 (beginning approximately 20 aa after the residue affectd by *dx99*) is required for association between channel subunits [52, 53]. Fujiwara et al. [54] performed structural studies demonstrating that the corresponding region of TRPM7 can form anti-parallel coiled-coils. Finally, Paulsen et al. showed that an overlapping ~80 aa region of TRPA1 forms parallel coiled coils that mediate association between channel subunits [25]. When analyzed by JPRED4 [44], this region of TRPA1 is predicted to be mostly helical, but with multiple interruptions. Significantly, JPRED4 predicts that an 88 amino acid segment of GON-2 that includes the site of *dx99* (NDQIEKLHDFEEDCMEDLARQKLNEKNTSNEQRILRADIRTDQILNRLIDLQAKESMGRDVINDVESRLASVEKAQNEILECVRALLN) is likely to be entirely helical except for a small gap (underlined residues). This is also true for TRPM6 and TRPM7, so this is likely to be a conserved structural feature that has the potential to mediate an extensive coiled-coil interaction between subunits. In summary, *dx99* could potentially affect *gon-2(q388)* activity by reducing ubiquitylation efficiency, altering affinity for regulatory phosphoinositides, or by potentiating successful channel assembly.

### Reversion of *gtl-2(0)*

*gtl-2(tm1463)* animals were mutagenized with EMS and then Mg^2+^-resistant revertants were selected as described [31]. In the case of one of the revertant mutations, *dx154*, we observed Gon animals at high frequency during outcrossing. Through standard mapping and complementation testing we determined that *dx154* was an allele of *gon-2*. Upon sequencing the coding reqions of *gon-2*, we found a single C->T mutation corresponding to *gon-2(dx154)*. This mutation is identical to *gon-2(dx87)*, which we identified in a previous screen for Gon mutants [36].

Two lines of evidence suggest that *gon-2(dx154)* is not a simple loss-of-function allele. First, the loss-of-function allel, *gon-2(q388)*, does not suppress the Mg^2+^ sensitivity of *gtl-2(tm1463)* mutants. Second, we isolated only a single allele of *gon-2* in this screen, whereas we identified multiple loss-of-function alleles of *gtl-1*, a gene of similar size.

The ability of *gon-2(dx154)* to suppress *gtl-2(tm1463)* can be explained most simply if *gon-2(dx154)* produces an antimorphic protein that is capable of interfering with GTL-1 activity. This is consistent with the tetrameric structure of TRPM channels, and supports that idea that GON-2 and GTL-1 are able to form heterotetramers.

#### Genetic characterization of *gon-2(dx87)*

We used a stock carrying the *gon-2(dx87)* mutation (identical to *dx154*) to test whether this allele is able to interfere with the activity of *gon-2(+)*. In order to do this, we examined the *gon-2(dx87)* homozygous progeny of *gon-2(dx87)/+* heterozygous hermaphrodites, comparing these with *gon-2(0)* (*ok465*) homozygous progeny from *gon-2(0)/+* hermaphrodites (Table 2, lines 6 and 7). Due to maternally contributed *gon-2(+)* gene product, *gon-2(0)* homozygotes are almost always able to execute a sufficient number of gonadal cell divisions to generate an anchor cell, and thus induce the underlying hypodermal cells to produce a vulva. However, 100% of *gon-2(dx87)* homozygous progeny of a heterozygous mother are vulvaless. Therefore, *gon-2(dx87)* is able to interfere with *gon-2(+)* in addition to *gtl-1(+)*. *gon-2(dx87)* is not strongly antimorphic, because *gon-2(dx87)/gon-2(+)* progeny of a *gon-2(dx87)* homozygous mother always undergo normal development (Table 2, line 3).

**Table 2:** Effects of *gon-2(dx87)* on *gon-2(+)* Activity and in Response to Different Divalent Cation Concentrations.

	Maternal Allele Configuration	Zygotic Allele Configuration	Mg^2+^	Ca^2+^	Gon	Evul	wt	n
1	*gon-2(dx87/gon-2(+)*	*gon-2(dx87/gon-2(dx87)*	0	0	95	4	1	81
2	*gon-2(ok465)/gon-2(+)*	*gon-2(ok465)/gon-2(ok465)*	0	0	3	12	85	103
3	*gon-2(dx87/gon-2(dx87)*	*gon-2(dx87/gon-2(+)*	0	0	0	0	100	151
4	*gon-2(dx87/gon-2(dx87)*	*gon-2(dx87/gon-2(dx87)*	0	0	100	0	0	253
5	*gon-2(dx87/gon-2(dx87)*	*gon-2(dx87/gon-2(dx87)*	1	0	0	3	97	177
6	*gon-2(dx87/gon-2(dx87)*	*gon-2(dx87/gon-2(dx87)*	10	0	0	0	100	425
7	*gon-2(dx87/gon-2(dx87)*	*gon-2(dx87/gon-2(dx87)*	1	10	89	5	5	340

Crossing schemes, full parental genotypes and culture conditions are described in Materials and Methods. Supplemental ion concentrations (in mM) are indicated. It should be noted that all strains were propagated on living *E*. *coli*, which contains at least trace amounts of Ca^2+^ and Mg^2+^, even when grown on divalent cation depleted medium.

One striking feature of *gon-2(dx87)* is that its effects on gonad development can be suppressed even by a low level of Mg^2+^ in the medium (1 mM; Table 2 lines 4–6). This is interesting, because it suggests that *gon-2(dx87)* homotetramers are able to permeate Mg^2+^ effectively into the gonad precursors. However, since *gon-2(dx154) (= dx87)* was isolated based on its ability to suppress the toxic hyperaccumulation of Mg^2+^ in *gtl-2(-)* animals that are grown on high Mg^2+^, heterotetrameric complexes that contain both GON-2(dx87) and GTL-1 are probably significantly less active than heteromeric complexes that contain only wild type proteins.

#### Possible mechanisms of action of *gon-2(dx87)*

**Fig 7:**
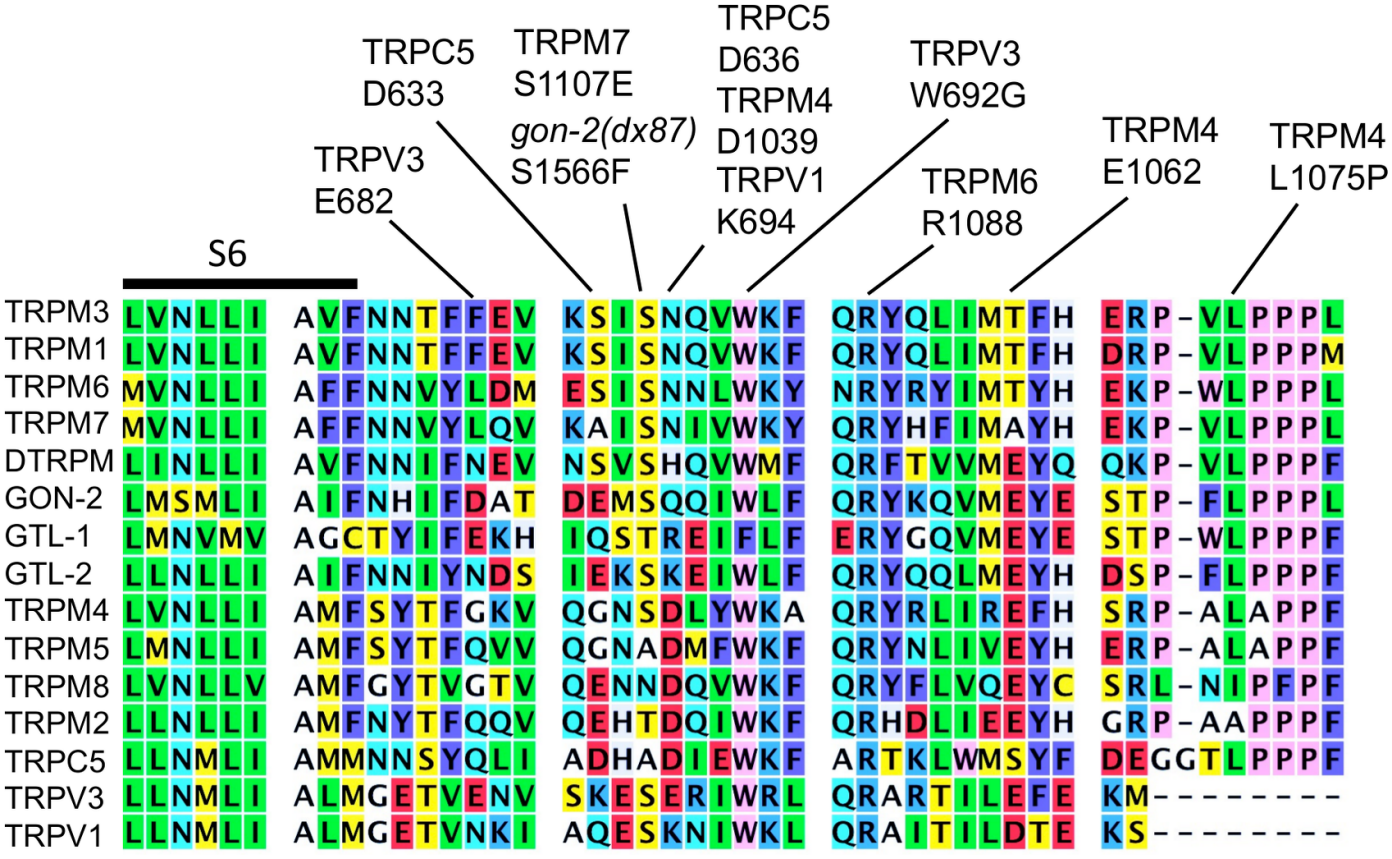
TRP protein alignment near *gon-2(dx87)*.

*dx87*converts a well-conserved serine within the TRP domain to a phenylalanine (Fig 7). Hofman et al. [55] showed that mutation of the corresponding serine residue of TRPM7 (S1107) to glutamate caused the channel to be constitutively active and insensitive to high concentrations (2 mM) of intracellular Mg^2+^. Similarly, Luo et al. showed that changing E682 of TRPV3 to either glutamine or asparagine caused the channel to be significantly less sensitive to inhibition by intracellular Mg^2+^ [56]. Furthermore, Lin et al. identified the W692G of TRPV3 mutation in patients with Olmsted syndrome and showed that this mutation causes the channel to be constitutively active [57]. They suggested that, as in voltage-gated potassium channels, the region immediately C-terminal to S6 can interact with the S4–S5 cytoplasmic loop to maintain a closed channel state. In the case of TRPC5, Obhukov et al. [20], found that D633 and D636 mediate channel blockage by intracellular Mg^2+^ at positive voltages. Although these are not physiological conditions, these data again suggest that Mg^2+^ may interact directly with the TRP domain to maintain a closed channel state, possibly by promoting interactions between the TRP domain and one or more other cytoplasmic segments (S4–S5 loop, pre-S1 helix).

Additional factors are also likely to interact with the TRP domain. For example, Xie et al. [58] showed that R1088 of TRPM6 R1088 is required for full activation by PI(4,5)P2. Two other basic residues nearby are also important, but these are not conserved in GON-2. Furthermore, in the case of TRPV1, Poblete found that K694 interacts with phospho-head group of PI(4,5)P2 [59]. Ca^2+^ probably also interacts with the TRP domain; Yamaguchi et al. showed that mutation of either E1062 or D1039 reduced the activation of TRPM4 by intracellular Ca^2+^, and argued that Ca^2+^ interacts directly with these residues [60]. It is also worth noting that Liu et al. found that the Brugada Syndrome L1075P allele of TRPM4 resulted in an increase in the amount of protein present on cell surface, suggesting a defect in endocytic trafficking [61].

Studies in *C*. *elegans*, indicate that GON-2 activity is regulated by each of the factors discussed above. Estevez et al. [45, 62] reported that outward currents through GON-2/GTL-1 (i.e., ORCa, [63]) are inhibited by submillimolar intracellular Mg^2+^, stimulated by relatively low intracellular Ca^2+^ (11 nM), and inhibited by higher levels of intracellular Ca^2+^ (250 nM). Based on steric considerations, they concluded that the likely site of inhibitory interaction with Ca^2+^ is in close proximity to the plasma membrane/channel pore, i.e, possibly within the TRP domain. In a subsequent study from the same group, Xing et al., [64] showed that PLC gamma activates GON-2/GTL-1, and that this effect probably results from a decrease in the concentration of PI(4,5)P2. Their results also indicated that the inhibitory effects of PI(4,5)P2 and Ca^2+^ on GON-2/GTL-1 are independent, and thus probably mediated by separate sites on the channel. In a separate study, Teramoto et al. [30] performed electrophysiological characterization of cells isolated from *gon-2(-)* and *gtl-1(-)* animals, as well as physiological experiments with single and double mutant animals, and thus were able to ascribe specific properties to the individual GON-2 and GTL-1 channels. Their key findings were that the GON-2 channel is inhibited by intracellular Mg^2+^ levels above 1 mM, whereas GTL-1 is not inhibited even at 6 mM intracellular Mg^2+^. *gon-2(-)* animals (which possess only GTL-1 activity) are relatively insensitive to growth inhibition by 10 mM Ca^2+^ in the medium, whereas *gtl-1(-)* mutants (which possess only GON-2 activity) grow very slowly under these conditions. Therefore, GON-2 is more sensitive than GTL-1 to inhibition by Ca^2+^. Furthermore, the growth defect of *gtl-1(-)* mutants on 0 mM supplemental Mg^2+^ could be rescued by 5 mM EGTA, which preferentially chelates Ca^2+^. It is unclear whether this inhibitory effect of Ca^2+^ is due to intracellular or extracelluar effects on GON-2.

One simple explanation for the effects of *gon-2(dx87)* would be that it destroys a residue that facilitates intracellular exit of divalent cations from the channel pore. This would be a logical consequence of the removal of the polar serine residue and/or introduction of the bulky hydrophobic phenylalanine residue. Alternatively, *gon-2(dx87)* could increase the affinity of the TRP domain for binding by a negative regulator, e.g., PI(4,5)P2, Mg^2+^ or Ca^2+^. Our data do not allow us to determine whether or not GON-2(dx87) is inhibited normally by intracellular Mg^2+^. However, GON-2(dx87) is clearly sensitive to inhibition by Ca^2+^, because the addition of 10 mM Ca^2+^ to medium that contains 1 mM Mg^2+^ causes an increase in the frequency of Gon animals from 0% to 89% (Table 2, line 7).

## Conclusions

In this study, we have identified seven evolutionarily conserved residues in the N-terminal cytoplasmic domain of GON-2, and one residue in the C-terminal cytoplasmic domain, as potentially important regulators of channel activity. Since each of these mutations was isolated as an intragenic revertant of the *gon-2(q388)* loss-of-function mutation, we do not know whether these mutations would affect channel activity when present in an otherwise wild type GON-2 protein. Furthermore, since our analyses are based on phenotypic output, we do not know the mechanism whereby these mutations affect channel activity, e.g., via altering protein folding, channel subunit trafficking, oligomeric assembly, or channel gating. We have also identified an unusual antimorphic allele of *gon-2* that converts a highly conserved serine residue within the TRP domain to phenylananine. It is particularly intriguing that this Hofman et al. [55] showed that changing the corresponding residue of TRPM7 to glutamate resulted in a channel that was consitutively active, even in the presence of high intracellular Mg^2+^. Therefore, this residue appears to be positioned at a key site involved in channel gating.
